# Annotation analysis for testing drug safety signals using unstructured clinical notes

**DOI:** 10.1186/2041-1480-3-S1-S5

**Published:** 2012-04-24

**Authors:** Paea LePendu, Srinivasan V Iyer, Cédrick Fairon, Nigam H Shah

**Affiliations:** 1 Stanford Center for Biomedical Informatics Research, Stanford University, USA; 2 Institut Langage et Communication, Centre de traitement automatique du langage, Université catholique de Louvain, Belgium

## Abstract

**Background:**

The electronic surveillance for adverse drug events is largely based upon the analysis of coded data from reporting systems. Yet, the vast majority of electronic health data lies embedded within the free text of clinical notes and is not gathered into centralized repositories. With the increasing access to large volumes of electronic medical data—in particular the clinical notes—it may be possible to computationally encode and to test drug safety signals in an active manner.

**Results:**

We describe the application of simple annotation tools on clinical text and the mining of the resulting annotations to compute the risk of getting a myocardial infarction for patients with rheumatoid arthritis that take Vioxx. Our analysis clearly reveals elevated risks for myocardial infarction in rheumatoid arthritis patients taking Vioxx (odds ratio 2.06) before 2005.

**Conclusions:**

Our results show that it is possible to apply annotation analysis methods for testing hypotheses about drug safety using electronic medical records.

## Background

### The case for post-approval drug safety

Adverse drug events currently result in significant costs: researchers estimate that adverse events occur in over 30% of hospital stays and 50% of these are drug-related events [1] that result in tens of billions of dollars in associated costs per year [2]. In 2004, Vioxx (rofecoxib) was taken off the market because of the increased risk of heart attack and stroke in patients who were taking the drug as a treatment for rheumatoid arthritis (RA) [3]. This case in particular generated public outcry and an appeal for better adverse drug event (ADE) detection mechanisms largely because Vioxx was on the market for four years despite murmurings of its side effects. In the past, Fen-Phen (fenfluramine/phentermine) was on the market with serious side effects for more than 24 years and resulted in one of the largest legal settlements ($14 billion) in US history [4].

To improve post-market drug safety, the U.S. Congress passed the U.S. Food and Drug Administration (FDA) Amendments Act of 2007, which mandated that the FDA develop a national system for using health care data to identify risks of marketed drugs and other medical products. The FDA subsequently launched the Sentinel Initiative in 2008 to create mechanisms that integrate a broader range of healthcare data and augment the agency’s current capability to detect ADEs on a national scale [5]. In related efforts, organizations like the Observational Medical Outcomes Partnership [6] have been established to address the use of observational data for active drug safety surveillance.

### Detecting adverse drug events

The current paradigm of drug safety surveillance is based on spontaneous reporting systems, which are databases containing voluntarily submitted reports of suspected adverse drug events encountered during clinical practice. In the USA, the primary database for such reports is the Adverse Event Reporting System (AERS) database at the FDA [7]. The largest of such systems is the World Health Organization’s Programme for International Drug Monitoring [8]. Researchers typically mine the reports for drug-event associations via statistical methods based on disproportionality measures, which quantify the magnitude of difference between observed and expected rates of particular drug-event pairs [9].

Partly in response to the biases [10] inherent in data sources like the AERS, or billing and claims databases, researchers are increasingly incorporating observational data directly from hospital electronic health record (EHR) databases [11-14] as well as published research from Medline abstracts to detect ADEs [15,16]. Recent advances on these methods include identifying combinations of drugs [17] that may lead to combinations of adverse events [12,18-21], and more closely address the real-life situation of patients taking multiple drugs concomitantly. Given advances in *detecting* (i.e., discovering or inferring) drug safety signals from the AERS, it becomes crucial to develop methods for *testing* (i.e. searching for or applying) these signals throughout the EHR.

### Gaps: mining clinical text and using terminologies

Despite the potential impact on improving patient safety, the full benefit of the EHR remains largely unrealized because the detailed clinical descriptions buried within the clinical text noted by doctors, nurses, and technicians in their daily practice are not accessible to data-mining methods [22-25]. Methods that rely on data encoded manually could be missing more than 90% of the adverse events that actually occur [1]. Fortunately, given advances in text processing tools [26-28], researchers can now computationally annotate and encode clinical text rapidly and *accurately enough *[29] to address real-world medical problems like ADE detection.

Using biomedical terminologies goes hand-in-hand with making the most of clinical text. Terminologies contain sets of strings for millions of terms that can be used as a lexicon to match against clinical text. Moreover, each terminology specifies relationships among terms and often includes a classification hierarchy. For example, the National Center for Biomedical Ontology (NCBO) BioPortal repository [30] contains about 300 terminologies and 5.4 million terms, including many from the Unified Medical Language System (UMLS) [31]. By linking patients and their clinical text to multiple terminologies via these lexical matches, researchers can make inferences that are not possible ordinarily when using a single classification hierarchy alone. Researchers could improve the predictive ability of surveillance efforts by making use of automated inference over drug families, diseases hierarchies, and their known relationships such as indications and adverse events for drugs [22]. For example, Baycol (cerivastatin), a drug for treating patients with high-cholesterol, was recalled in 2001 for increased risk of rhabdomyolysis, a muscle disorder that can lead to kidney failure and possibly death. By reasoning over the known relationship between myopathy and rhabdomyolysis that is encoded in standard biomedical terminologies like MedDRA and SNOMED-CT, researchers could have automatically inferred the adverse relationship between myopathy and cerivastatin and prevented 2 years of unmitigated risk for other patients [9]. In other words, terminologies can make it possible to integrate and to aggregate resources automatically not only by providing a lexicon of terms from many different vocabularies, but also by assimilating information at different levels of specificity among those vocabularies.

We hypothesize that simple methods can be used to annotate and to mine the clinical text of a large number of patients for testing drug safety signals. To validate this hypothesis, we tested a well-known signal by annotating the clinical text of more than one million patients from the Stanford Clinical Data Warehouse (STRIDE) and computing the risk of getting a myocardial infarction for rheumatoid arthritis patients who took Vioxx [3].

## Results

**Figure 1:**
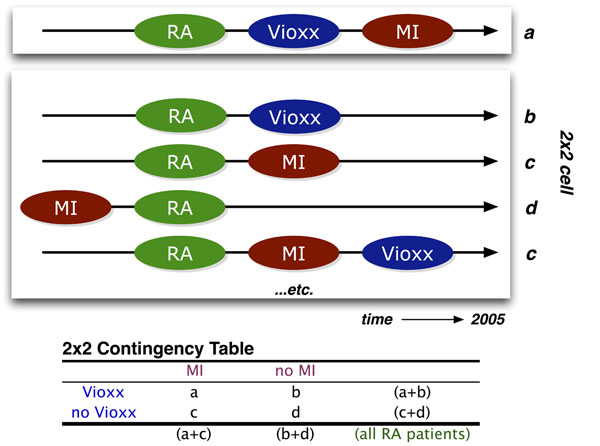
**The NCBO Annotator Workflow** extracts terms from the clinical notes of patients: (1) We obtain a lexicon of over 2.8-million terms from the NCBO BioPortal library. (2) We use the NCBO Annotator to rapidly find those terms in clinical notes—which we call annotations. (3) We apply NegEx trigger rules to separate negated terms. (4) We compile terms (both positive and negative) into a temporally ordered series of sets for each patient and combine them with coded and structured data when possible. (5) We reason over the structure of the ontologies to normalize and to aggregate terms for further analysis.

Graham et al. showed that patients having rheumatoid arthritis (RA) who took Vioxx (rofecoxib) showed significantly elevated risk (odds ratio=1.34) for myocardial infarction (MI), which resulted in the drug being taken off the market in 2004 [3]**.** To reproduce this risk, we identify patients in the EHR who have the given condition (RA), who are taking the drug, and who suffer the adverse event (Figure 1) by using automated annotation analysis. We call each condition–drug–event being tested a hypothesized drug safety signal “triplet.”

Our main result is that the annotated notes clearly show an elevated risk for Vioxx. In addition, the risk is not present using ICD-9 discharge codes alone, which demonstrates the importance of using the notes for testing drug safety signals. The key feature of our analysis that differentiates our methods from all others that we know about is the simplicity and speed of our annotation workflow, which can be installed in minutes and run overnight on an entire clinical data warehouse having millions of patients. Moreover, once the annotation data is compiled, it takes 10 seconds to test a single signal. To demonstrate the point that we can enable testing drug safety signals in general—on a massive scale—as a result of this workflow, we also recapitulate two other well-known signals and report those results below as well. We have also tested over 10,000 other triplets (some known signals, some hypothesized signals, and some chosen at random)—those results will be validated and reported as separate works.

### Annotating 9 million clinical notes overnight

We extract data on the conditions, drugs, and events present in over one million patients’ histories directly from their clinical reports by using an automated annotation workflow. The annotation workflow is packaged on a USB stick and can be installed on a basic Linux machine (4 CPUs, 16GB RAM, 50GB hard disk) in minutes to run within the HIPAA secured environment of the clinical data warehouse. Overnight, in approximately 7 hours (a little more than 1 million notes per hour), the annotator will have matched a lexicon of over 2.8 million terms to all 9 million clinical notes. It returns a compressed, de-identified patient feature matrix having 9 million rows (one for every clinical note) and 2.8 million columns, which is small enough to be saved back on the USB stick for analysis outside the secure environment. It takes approximately 12 hours of post-processing to normalize and index the annotation data for analysis (also on a single, basic Linux machine). After using the ontologies to normalize the terms, the number of columns in the patient feature matrix reduces to the order of 30,000 clinical concepts, which makes analysis more tractable.

### Identifying patients of interest

From the full patient feature matrix, the subset of all rows matching either a condition, drug, or event of interest constitutes the set of all patient notes relevant for testing a drug safety signal. The rows, which denote clinical notes, are sorted by patient and then by time. The sorted rows essentially constitute a timeline-view for all patients of interest. To recapitulate the Vioxx risk signal, we constructed a timeline-view for all patients matching RA, Vioxx, or MI at any point in time, which consists of 550,000 rows (notes) for 154,000 patients. This process takes anywhere between 5-30 seconds depending on how many patients are matched.

### Constructing the Vioxx contingency table

**Figure 2:**
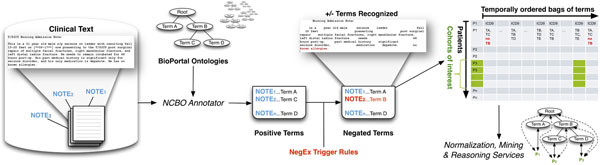
The Vioxx risk pattern (top row) occurs when a patient with rheumatoid arthritis (RA) who takes Vioxx and suffers a myocardial infarction (MI)—these frequencies are entered into cell *a* of the 2x2 contingency matrix. Based on the various combinations of temporal orderings for the initial mentions of RA, Vioxx, and MI in the patient notes, other possible patterns contribute to the expected background distribution (cells *b*, *c*, *d* of the contingency table) that the odds ratio calculation requires.

From the timeline-view of all patients matching any portion of the Vioxx triplet, we remove all records before 1999 and after 2005, since Vioxx was only on the market during that period. From the various permutations of clinical concepts for each patient history (e.g., having RA, Vioxx and MI follow one another in time, versus having RA but never Vioxx or never MI), we simply count the number of patients for each pattern (see Figure 2) and add the counts to the contingency table shown in Table 1 appropriately. These temporal filters reduce the set of patients from 154,000 to just over 14,000. From the contingency table, we calculate the odds ratio (OR) and significance as described in Methods (Table 4) [9].

**Table 1:** Two-by-two contingency table for rofecoxib and myocardial infarction within the STRIDE dataset for patients with rheumatoid arthritis before 2005 using NCBO annotations.

	myocardial infarction	no myocardial infarction
rofecoxib	a=339	b=1221
no rofecoxib	c=1488	d=11031

**Table 2:** Two-by-two contingency table for rofecoxib and myocardial infarction within the STRIDE dataset for patients with rheumatoid arthritis before 2005 using mainly ICD9 coded data.

	myocardial infarction	no myocardial infarction
rofecoxib	a=16	b=487
no rofecoxib	c=61	d=2831

**Table 3:** Ontologies chosen for Annotation Workflow configuration as well as overall term frequency counts per ontology.

*Ontology Name*	*Source*	*Abbreviation*	*Frequency*
Current Procedural Terminology	UMLS	CPT	17243153
Human Disease Ontology	OBO	DO	122035173
International Classification of Disease (ICD-10)	UMLS	ICD10	55572189
International Classification of Disease (ICD-9)	UMLS	ICD9	58334369
Logical Observation Identifier Names and Codes	UMLS	LNC	1208284117
Medical Dictionary for Regulatory Activities	UMLS	MDR	361398956
Medical Subject Headings	UMLS	MSH	643026014
National Drug File	UMLS	NDFRT	232557746
NCI Thesaurus	UMLS	NCI	2498591490
Online Mendelian Inheritance in Man	UMLS	OMIM	262747872
Systematized Nomenclature of Medicine–Clinical Terms	UMLS	SNOMEDCT	2369959351

**Table 4:** Odds Ratio: For a given pair (x,y), the reporting odds ratio provides the “unexpectedness of y, given x” via the simple calculation (ad) ÷ (bc) as defined in the contingency table below. For example, the cell marked as *a* denotes the number of patients that took drug *x* (e.g., Vioxx), and experienced condition *y* (e.g., myocardial infarction). The other cells help to establish the likelihood.

	y	not y
**x**	a	b
**not x**	c	d

### Significant Vioxx risk signal in clinical text

Mentions of Vioxx (rofecoxib) demonstrate a significantly associated risk for myocardial infarction (MI) in patient clinical notes mentioning rheumatoid arthritis (RA) before 2005. Analysis of the 2x2 contingency matrix (Table 1) for the association between rofecoxib and MI results in an odds ratio of 2.06 with confidence interval 1.80–2.35 and p-value less than 10^-7^ using Fisher’s exact test (two-tailed). This confirms an elevated risk of having mentions of myocardial infarction follow mentions of Vioxx. This result confirms the known risk for Vioxx.

### No significant Vioxx risk signal in ICD-9 discharge diagnosis codes

In contrast, using coded discharge diagnoses (ICD9 codes) without any clinical text, the same patient records demonstrate no significant risk—odds ratio is 1.52 with confidence interval 0.87–2.67 and p-value 0.19 (Table 2). The ICD-9 codes for all patient visits were extracted from the data separately to make this comparison. This result demonstrates clearly the importance of using data buried in the clinical notes.

### Testing other known signals

In addition to testing for the *rheumatoid arthritis–Vioxx–myocardial infarction* signal, we also tested the signals for *diabetes–Actos–bladder neoplasm* (odds ratio 1.51, p-value < 10^-7^), and *hypercholesterolemia–Baycol–rhabdomyolysis* (odds ratio 7.65, p-value 2.05x10^-4^). Recapitulating these known signals further confirms the efficacy and importance of using data buried in the clinical notes.

## Discussion

Our results confirm that it is possible to validate risk signals for some of the most controversial drugs in recent history by analyzing annotations on clinical notes. Furthermore, our methods are easy to deploy within the protected HIPPA environments of most hospitals, our methods produce de-identified data that can be exported and analyzed on more robust platforms if necessary, and our methods scale to millions of patients without any complicated infrastructure or configuration.

### Term extraction versus natural language processing

Clearly, our results hinge upon the efficacy of the annotation mechanism. We have conducted a comparative evaluation of two concept recognizers used in the biomedical domain—Mgrep and MetaMap—and found that Mgrep has clear advantages in large-scale, service-oriented applications specifically addressing flexibility, speed and scalability [28]. The NCBO Annotator uses Mgrep. The precision of concept recognition varies depending on the text in each resource and type of entity being recognized: from 87% for recognizing disease terms in descriptions of clinical trials to 23% for PubMed abstracts, with an average of 68% across four different sources of text. We are currently conducting evaluations in collaboration with outside groups and early results from researchers at the University of Pittsburgh (Richard Boyce, personal communication) show that using RxNORM gives 93% recall for detecting drug mentions in clinical text. For the current work, we assume a similar level of performance. In the future we will manually examine samples of patient records to validate the ability to recognize diseases in clinical notes. We have previously employed such sampling based strategies to evaluate the accuracy of annotation workflows applied to very large datasets [32].

Our goal is to explore methods that work for detecting signals at the population-level, and not necessarily at an individual level. Therefore, in contrast with using a full-featured natural language processing (NLP) tool, our goal is to develop simple, fast, and *good-enough* term recognition methods that can be used on very large datasets. To the best of our knowledge, NLP tools do not function at the scale of tens of millions of clinical notes. However, when they do reach the necessary level of scalability, we can use their more advanced capabilities to improve our methods. In the meantime, we have begun to include contextual cues (e.g., family history) by incorporating tools like ConText [33] as a means of improving the precision with which we determine whether drug is prescribed or a disease is diagnosed. We are also investigating regular expression based tools like Unitex [34] that demonstrate the kind of speed and scalability required while adding more powerful pattern recognition features like morpheme-based matching.

### False discovery rates in signal detection

Given the level of noise we might expect with automated annotation, signal detection as we have described remains quite robust. For example, cell *a* in the contingency table (Table 4) must be as accurate as possible. Assuming a 20% false positive rate, the likelihood of getting cell *a* wrong is very low (0.8%) because we would need to over-estimate all three annotations at the same time, which is very unlikely. Adjusting all cells in the 2x2 table for a 20% false positive rate still yields a significant odds ratio of 1.43 (confidence interval 1.21–1.68, p-value 4.3x10^-5^). On the other hand, ICD9 coding likely results in no signal in our dataset because it severely underestimates the actual likelihood—a patient who has RA may only get coded for treating, say, an ulcer because of the nature of the billing and discharge diagnosis mechanism, but notes on their history will clearly show that they have RA. Therefore, we are still reasonably confident that we are seeing true signals despite some degree of noise.

Another disproportionality measure that we have explored is the use of enrichment analysis techniques adapted from high-throughput analysis of genes [35]. What makes the use of enrichment analysis interesting is that the use of ontologies and the handling of false discovery rates is well studied [36]. As with using propensity score adjustments [37], one of the key issues is to choose an appropriate background distribution from which to infer that an unlikely scenario has occurred. In some cases, researchers use a control group, such as patients having minor complications [38]. In our case, we limit the background by restricting the cohort to patients with the given indication (e.g., RA).

Given that we have successfully tested three well-known drug safety signals, the focus of our ongoing work will be to detect *new* drug safety signals given patterns that have not yet been reported. Two key challenges we are currently addressing include: 1) controlling for the false discovery rate of new signals, 2) prioritizing new signals worth testing. In addition to manually reviewing patient records for annotation accuracy, we will also be using a combination of data sources like AERS and the Medicare Provider Analysis and Review data for cross-validation. To prioritize signals worth testing, we have begun investigating methods to automatically generate “triplets” (e.g., *RA-Vioxx-MI* ) for drugs that are used frequently, have inadequate supporting evidence such as drugs used off-label [39], and that are likely to be associated with severe adverse events [40].

### The role of ontologies and mappings

Ontologies play two vital roles in our workflow: 1) they contribute a vast and useful lexicon; 2) they define complex relationships and mappings that can be used to enhance analysis. Although using ontologies for normalization and aggregation are clearly beneficial, they also present a key challenge that arises when using large and complex ontologies, let alone many of them simultaneously. The challenge is to determine which abstraction level to use for reporting results. In enrichment analysis, this issue has been well studied with respect to the Gene Ontology [36] and the methods have been extended to use other ontologies [35]. For drugs, it may be obvious to normalize all mentions to either active ingredients or generics. However, with diseases and conditions, it is not always clear what level of abstraction makes the most sense for any given analysis. For example, counting patients with *bladder papillary urothelial carcinoma* as persons with *bladder cancer* in the Actos study is probably more useful than aggregating up to the level of *urinary system disorder*. But if we also want to know what diseases are most frequently co-morbid with patients having bladder cancer, then the sheer number of related diseases and all of their more specific kinds creates an exponential explosion of combinations to consider. To address this problem, we are investigating the use of information theory—specifically, information content—to partition the space of possible abstraction levels into bins that represent similar levels of specificity across the board [41], which should make the aggregation of results at similar levels of specificity more tractable.

## Conclusions

We analyzed the automatically created annotations on clinical notes and recapitulated three different drug safety signals. We found that the risk is far more perceptible when the unstructured data in the EHR is used versus using coded data alone. We recapitulated the risk by means of the odds ratio—a widely used disproportionality metric—applied to annotations of unstructured clinical text and demonstrated the potential for annotation analysis methods.

Our results establish the feasibility of using annotations created from clinical notes as a source for testing as well as possibly detecting drug safety signals. We believe that analysis of the clinical text can be used to bolster detection mechanisms for finding serious side effects of post-market drugs and thus improve patient safety.

## Methods

### Clinical data

We use data from the Stanford Clinical Data Warehouse (STRIDE), which is a repository of 17-years worth of patient data at Stanford. It contains data from 1.6 million patients, 15 million encounters, 25 million coded ICD9 diagnoses, and a combination of pathology, radiology, and transcription reports totalling over 9.5 million unstructured clinical notes. After filtering out patients to satisfy HIPAA requirements (e.g., rare diseases, celebrity cases, mental health), we annotated 9,078,736 notes for 1,044,979 patients. The gender split is roughly 60% female, 40% male. Ages range from 0 to 90 (adjusted to satisfy HIPAA requirements), with an average age of 44 and standard deviation of 25.

### Annotating clinical texts

We created a standalone Annotator Workflow (Figure 1) based upon the existing National Center for Biomedical Ontology (NCBO) Annotator Web Service [28] that annotates clinical text from electronic health record systems and extracts disease and drug mentions from the EHR. Unlike natural language processing methods that analyze grammar and syntax, the Annotator is mainly a term extraction system: it uses biomedical terms from the NCBO BioPortal library and matches them against input text. We have also extended the Annotator Workflow by incorporating the NegEx algorithm [42] to incorporate negation detection—the ability to discern whether a term is negated within the context of the narrative. We are also extending the system to discern additional contextual cues [33] such as family history versus recent diagnosis.

One strength of the Annotator is the highly comprehensive and interlinked lexicon that it uses. It can incorporate the entire NCBO BioPortal ontology library of over 250 ontologies to identify biomedical concepts from text using a dictionary of terms generated from those ontologies. Terms from these ontologies are linked together via mappings [30]. For this study, we specifically configured the workflow to use a subset of those ontologies (Table 3) that are most relevant to clinical domains, including Unified Medical Language System (UMLS) terminologies such as SNOMED-CT, the National Drug File (NDFRT) and RxNORM, as well as ontologies like the Human Disease Ontology. The resulting lexicon contains 2.8 million unique terms.

Another strength of the Annotator is its speed. We have optimized the workflow for both space and time when performing large-scale annotation runs. It takes about 7 hours and 4.5 GB of disk space to process 9 million notes from over 1 million patients. Furthermore, the entire system fits on a USB stick and takes 45 minutes to configure and launch on most systems. To the best of our knowledge, existing NLP tools do not function at this scale.

The output of the annotation workflow is a set of negated and non-negated terms from each note (Figure 1, step 3). As a result, for each patient we end up with a temporal series of terms mentioned in the notes (red denotes negated terms in Figure 1, step 4). We also include manually encoded ICD9 terms for each patient encounter as additional terms. Because each encounter’s date is recorded, we can order each set of terms for a patient to create a timeline view of the patient’s record. Using the terms as features, we can define patterns of interest (such as patients with *rheumatoid arthritis*, who take *rofecoxib*, and then get *myocardial infarctio*), which we can use in data mining applications.

### Normalizing and aggregating terms

We use the RxNORM terminology to normalize the drug having the trade name Vioxx into its primary active ingredient, rofecoxib. From the set of ontologies we use, the Annotator identifies all notes containing any string denoting this term as either its primary label or synonym. We use all other ontologies to normalize strings denoting rheumatoid arthritis or myocardial infarction and the Annotator identifies all notes containing them.

As an option, we can also enable reasoning to infer all subsumed terms, which increases the number of notes that we can identify beyond pure string matches. For example, patients with Caplan’s or Felty’s syndrome may also fit the cohort of patients with rheumatoid arthritis. Therefore, notes that mention these diseases can automatically be included as well—even though their associated strings look nothing alike. We did not use such reasoning for results reported in this specific study.

### Obtaining ICD9 discharge codes

Patient visits include in some cases the discharge diagnosis in the form of an ICD9 code. The ICD9 codes for rheumatoid arthritis begin with 714 and the ICD9 code for myocardial infarction begins with 410. We include these manually encoded terms as part of the analysis as a comparison against what we can find in the text itself.

### Computing the risk of adverse drug events

The odds ratio (OR) is one measure commonly used to estimate the relative risk of drug adverse events [9]. The ratio gives one measure of disproportionality—the unexpectedness of a particular association occurring given the all other observations. The method for calculating the OR is summarized in Table 4.

We adopt the OR measure to infer the likelihood of an outcome like myocardial infarction when we compare the population exposed to a drug like Vioxx versus those who are not exposed. Rather than use the entire set of one million patients as the background population, we restrict the analysis to the subset of patients who demonstrate the usual indication, which for Vioxx would be rheumatoid arthritis. Applying this restriction ensures that patients who have zero propensity to be exposed to Vioxx do not get included in the analysis and thus avoids biasing the result [37].

We consider patients to be exposed (cells *a* and *b* in Table 4) only if their record demonstrates that the very first mention of Vioxx follows a mention of rheumatoid arthritis based on the ordering of timestamps for the notes in which the terms were found. The idea is that the patient should most likely be receiving Vioxx as a treatment for arthritis. Likewise, we consider patients to be experiencing the adverse event (cells *c* and *d* in Table 4) if myocardial infarction follows mentions of arthritis. Finally, those patients who potentially get myocardial infarction as a result of taking Vioxx (cell *a* in Table 4) requires that myocardial infarction follows Vioxx (which follows arthritis) in the notes. Figure 2 illustrates several of the patterns of interest that contribute to each cell of the contingency table.

## Competing interests

The authors declare there are no competing interests.

## Authors’ contributions

NHS led the study and provided research mentoring to PL and SVI. PL designed and executed the study, and directed SVI’s efforts. SVI and PL worked together to implement the algorithms necessary for extracting lexicons, producing annotations, and analyzing results. CF designed an alternative annotation workflow based on Unitex and suggested using ICD9 codes as a comparison metric.
